# Bleeding risk between newer direct-acting oral anticoagulants and selective serotonin reuptake inhibitors. Case report and literature review

**DOI:** 10.1192/j.eurpsy.2021.1293

**Published:** 2021-08-13

**Authors:** T. Gutiérrez Higueras, F. Calera Cortés, S. Sainz De La Cuesta Alonso, S. Vicent Forés

**Affiliations:** Clinical Unit Of Mental Health., Reina Sofia University Hospital., Córdoba., Spain

**Keywords:** selective serotonin reuptake inhibitors, Atrial Fibrillation, anticoagulants, bleeding risk

## Abstract

**Introduction:**

The use of selectiveserotonin reuptake inhibitors (SSRIs) is an independent risk factor for bleeding events. Antidepressants and oral anticoagulants (OACs) are often prescribed together as depression and anxiety often coexist with cardiovascular diseases, atrial fibrillation and thromboembolic disorders. Serotonin is released from platelets in response to vascular injury, promoting aggregation. Inhibition of serotonin transporter (responsible for the uptake of serotonin into platelets) can lead into a reduced ability to form clots and a subsequent increase in the risk of bleeding. Direct oral aticoagulants (DOACs), rivaroxaban, apixaban and edoxaban are primarily metabolized via CYP3A4. The co-administration of antidepressants with inhibitory effects on CYP3A4 may theoretically interact with them.

**Objectives:**

Presentation of a case of upper gastrointestinal bleeding after initiation of Apixaban in a patient taking Sertraline and literature review.

**Methods:**

We carried out a literature review in Pubmed electing those articles focused on bleeding risk between newer direct oral antigulants and selective serotinin reuptake inhibitors.

**Results:**

A 66-year-old woman sought medical assistance for generalized ecchymosis and melena. She was diagnosed with atrial fibrillation treated with apixaban 7 days ago. Concomitant treatment between apixaban and sertraline was the possible cause of upper gastrointestinal bleeding and ecchymosis. We had to switch sertraline into vortioxetine (with less dregree of serotonin reuptake inhibition) and add proton-pump inhibitor (Omeprazole) in order to decrease the risk of bleeding.

**Conclusions:**

SSRIs increase the risk of gastrointestinal bleeding, much more in case of concomitant use of oral anticoagulants. If SSRI use cannot be avoided, monitor closely and prescribe proton pump inhibitors.

